# Design effect in multicenter studies: gain or loss of power?

**DOI:** 10.1186/1471-2288-9-39

**Published:** 2009-06-18

**Authors:** Emilie Vierron, Bruno Giraudeau

**Affiliations:** 1 INSERM, CIC 202, Tours, France; 2 Université François-Rabelais de Tours, Tours, France; 3 CHRU de Tours, Tours, France; 4 INSERM, U717, Paris, France

## Abstract

**Background:**

In a multicenter trial, responses for subjects belonging to a common center are correlated. Such a clustering is usually assessed through the design effect, defined as a ratio of two variances. The aim of this work was to describe and understand situations where the design effect involves a gain or a loss of power.

**Methods:**

We developed a design effect formula for a multicenter study aimed at testing the effect of a binary factor (which thus defines two groups) on a continuous outcome, and explored this design effect for several designs (from individually stratified randomized trials to cluster randomized trials, and for other designs such as matched pair designs or observational multicenter studies).

**Results:**

The design effect depends on the intraclass correlation coefficient (ICC) (which assesses the correlation between data for two subjects from the same center) but also on a statistic *S*, which quantifies the heterogeneity of the group distributions among centers (thus the level of association between the binary factor and the center) and on the degree of global imbalance (the number of subjects are then different) between the two groups. This design effect may induce either a loss or a gain in power, depending on whether the *S *statistic is respectively higher or lower than 1.

**Conclusion:**

We provided a global design effect formula applying for any multicenter study and allowing identifying factors – the ICC and the distribution of the group proportions among centers – that are associated with a gain or a loss of power in such studies.

## Background

Multicenter studies involve correlation in data because subjects from the same center are more similar than are those from different centers [1]. Such a correlation potentially affects the power of standard statistical tests, and conclusions made under the assumption that data are independent can be invalidated.

A usual measure of the clustering effect on an estimator (often a treatment or a group effect) is the design effect (*Deff*). The *Deff *is defined as the ratio of two variances: the variance of the estimator when the center effect is taken into account over the variance of the estimator under the hypothesis of a simple random sample [2,3]. The *Deff *represents the amount by which the sample size needs to be multiplied to account for the design of the study. Ignoring clustering can lead to over- (*Deff *< 1) or underpowered (*Deff *> 1) studies.

In cluster randomized trials, clustering produces a loss of power and Donner and Klar proposed a method to inflate the sample size to take data correlation into account [4]. On the contrary, in individually randomized trials with equal treatment arm sizes, a center effect induces a gain in power, and sample size can be reduced [5]. Thus, in some situations, correlation in data induces a loss of power, and in others, a gain in power. To our knowledge, complete explanations for this striking discrepancy are lacking.

We aimed to produce a measure of clustering in multicenter studies testing the effect of a binary factor on a continuous outcome. We first present the statistical model used and the associated design-effect formula. Then we explore the general form of this design effect under particular study designs. Finally, we give examples to illustrate our results.

## Methods and results

### Theoretical Issues

#### The mixed-effects model

Let us consider a multicenter study aimed at comparing two groups on a continuous outcome. Several situations can be considered. If subjects are randomly assigned to a group (e.g., a treatment arm), the study is a randomized trial; otherwise, it is an observational study, and the group data depicts exposure to a binary risk factor. Data are distributed as follows:(1)

where *Y*_*ijk *_denotes the response from the *k*th subject, of the *i*th group, in the *j*th center. The overall response mean is *μ*. Each center is of size *m*_*j *_= *m*_1*j *_+ *m*_2*j*_, and each group is of size , with *N *= *n*_1 _+ *n*_2 _being the total number of subjects in the study. The group effects {*α*_*i*_} are fixed, with . We assume that centers are a random sample of a large population of centers, so the center effects {*B*_*j*_} are independent and identically distributed (iid) . The residual errors {*ε*_*ijk*_} are assumed to be  and independent of {*B*_*j*_}. The center effect, quantified by the intraclass correlation coefficient (ICC), *ρ*, and defined as the proportion of the total variance that is due to the between-center variability, can be defined from model (1) as follows [6]:(2)

#### Group effect variance

##### Two-way ANOVA

The group effect variance can be shown to equal (Appendix 1):(3)

##### One-way ANOVA

Ignoring the center effect, model (1) reduces to:(4)

where *Y*_*ik *_represents the response from the *k*th subject in the *i*th group. The random errors {} are iid . Thus, the variance of the group effect is as follows:(5)

and we have (Table 1):(6)

**Table 1:** One-way ANOVA for data distributed according to the two-way mixed-effects model (1).

Source	DF	SS	E(MS)
Group	2 - 1		
Residual	*N *- 2		

Total	*N *- 1		

When data are distributed according to the mixed model (1) but analyzed by performing a one-way ANOVA – as if data were distributed according to model (4) – the expectation of the residual mean squares (denoted  in the framework of model (4)) can actually be expressed as a function of  and , the variance components associated to the true underlying statistical model (i.e. the mixed model (1)).

#### The Design Effect

The *Deff *measures the effect of clustering on the group effect variance. It is defined as the ratio of the group effect variances (3) over (5). Using equation (6) we have:(7)

Multicenter randomized trials often recruit a large number of subjects. Then, assuming a large total sample size and numerous centers, the {*m*_*ij*_} are small in comparison with *N*, and  can be approximated by 1. Expression (7) then becomes:(8)

where *ρ *is the ICC as defined in (2) and .

#### Simulation study

We first conducted a simulation study aiming validating the approximate formula we proposed. We considered equal and varying center sizes for 12 combinations of the total sample size and number of centers (100 subjects for 5, 10 or 20 centers, 200 subjects for 5, 10, 20 or 50 centers, 500 subjects for 5, 10, 20, 50 or 100 centers), 4 group distributions (from balanced groups within centers to randomization of centers, which are then nested within the groups) and two ICC values (0.01 and 0.10). One thousand simulations were conducted using SAS 9.1 (SAS Institute, Cary, NC) for each combination of the parameters. Table 2 presents the average exact design effect estimate and average relative difference between exact and approximate design effect calculations for all these situations, for varying center sizes (20% of centers recruit 80% of subjects). Although such extreme imbalance in center sizes is unlikely to occur (and not advisable, mainly in cluster trial designs including very few centers, such as 5 or 10 centers), it allows testing the robustness of our formula even in such extreme situations. Similar results were found for equal center sizes (data not shown). Results show that the approximate design effect formula always slightly underestimates the exact formula since all relative differences are positive. These differences increase with the ICC and decrease, as expected, while the number of centers increases but are not influenced by the total number of subjects. Moreover, they globally increase with the design effect. All of these results are below (or equal) 0.0771, indicating that our formula applies in the majority of multicenter designs, with a better accuracy (relative differences lesser than 0.052) for designs including more than 10 centers.

**Table 2:** Validation of the approximate design effect formula.

ICC = 0.01												
N subjects	100			200				500				
N centers	5	10	20	5	10	20	50	5	10	20	50	100

S1 *Deff*	0.9969	0.9938	0.9921	0.9966	0.9936	0.9922	0.9911	0.9965	0.9933	0.9919	0.9913	0.9908
*rdiff*	0.0065	0.0032	0.0016	0.0065	0.0032	0.0016	0.0006	0.0065	0.0032	0.0016	0.0006	0.0003

S2 *Deff*	0.9972	0.9949	0.9928	0.9972	0.9956	0.9938	0.9917	0.9980	0.9989	0.9956	0.9931	0.9918
*rdiff*	0.0065	0.0032	0.0014	0.0065	0.0032	0.0016	0.0005	0.0065	0.0033	0.0016	0.0006	0.0003

S3 *Deff*	1.0102	1.0306	1.0147	1.0217	1.0622	1.0431	1.0132	1.0575	1.1788	1.1143	1.0487	1.0204
*rdiff*	0.0066	0.0035	0.0016	0.0066	0.0036	0.0018	0.0006	0.0066	0.0036	0.0019	0.0007	0.0003

S4 *Deff*	1.1038	1.0323	1.0285	1.2026	1.0538	1.0604	1.0184	1.4788	1.1290	1.1588	1.0559	1.0186
*rdiff*	0.0077	0.0051	0.0027	0.0077	0.0052	0.0030	0.0011	0.0077	0.0053	0.0030	0.0013	0.0006

												

ICC = 0.10												

N subjects	100			200				500				
N centers	5	10	20	5	10	20	50	5	10	20	50	100

S1 *Deff*	0.9655	0.9356	0.9197	0.9642	0.9337	0.9209	0.9105	0.9631	0.9313	0.9177	0.9124	0.9076
*rdiff*	0.0643	0.0318	0.0155	0.0649	0.0320	0.0160	0.0061	0.0649	0.0324	0.0161	0.0063	0.0031

S2 *Deff*	0.9709	0.9469	0.9269	0.9696	0.9547	0.9359	0.9171	0.9793	0.9827	0.9549	0.9300	0.9174
*rdiff*	0.0656	0.0318	0.0142	0.0648	0.0323	0.0157	0.0053	0.0651	0.0325	0.0161	0.0063	0.0028

S3 *Deff*	1.1101	1.3018	1.1721	1.2095	1.6471	1.4256	1.1337	1.6662	2.7175	2.1685	1.4965	1.2049
*rdiff*	0.0654	0.0349	0.0166	0.0659	0.0354	0.0182	0.0063	0.0662	0.0358	0.0185	0.0074	0.0034

S4 *Deff*	2.0718	1.3360	1.2725	3.1669	1.5750	1.6252	1.1934	6.2708	2.5759	2.5886	1.5687	1.2017
*rdiff*	0.0768	0.0507	0.0272	0.0770	0.0517	0.0299	0.0110	0.0771	0.0513	0.0299	0.0126	0.0059

ICC: Intraclass Correlation Coefficient

Simulations are conducted with varying center sizes: 20% of centers recruit 80% of subjects. Average exact design effect estimate (*Deff*) and average relative difference (*rdiff*) between exact and approximate design effect formula are given for 4 situations (Si, i = 1,2,3,4), two ICC values, and obtained for 1000 simulations.

S1: Equal group sizes. In each center, the probability, for a subject, to be in group 1 is 1/2

S2: Slight variations in group 1 proportions among centers. The ratio between the sizes of group 1 and group 2 varies uniformly between 0.8 and 1.25 among centers

S3: Important variations in group 1 proportions among centers. The ratio between the sizes of group 1 and group 2 varies uniformly between 0.1 and 10 among centers

S4: "Cluster design". The center is nested within the group and the probability, for each center, to be in group 1 is 1/2

#### Some specific designs

##### Stratified Multicenter Individually Randomized Trial

Assuming that randomization is balanced and stratified on centers, we then have equal group size () and equal number of subjects in the two groups in each center (∀ *j *= 1,..., *Q*, ). The *Deff *reduces to:(9)

In a stratified multicenter individually randomized trial, the *Deff *is smaller than 1 and its value decreases as the ICC increases, which involves a gain in power allowing a reduction in sample size, as shown by Vierron *et al*. [5].

##### Matched Pair Design

Some studies yield observations that are individually matched, such as cross-over trials, trials on matched subjects (which are, for example, matched by age or sex) or data (*e.g*. two eyes from the same subject) or before-after studies. Assuming pairs of matched data, pairs can be considered as centers, thus leading to a particular case of the stratified multicenter individually randomized trial with *m*_1*j *_= *m*_2*j *_= 1. Then the *Deff *equals:(10)

In a matched pair design, the variance of the differences between paired responses equals:(11)

where *σ*^2 ^is the variance of observations in a standard parallel group design.

Then, correcting the classical sample size formula for two independent samples with the *Deff *(1 - *ρ*) and replacing the *σ*^2^(1 - *ρ*) term by  leads to the sample size formula used for paired data studies [7]:(12)

where *d *is the difference in mean responses from the two groups.

##### Cluster Randomized Trial and Expertise-based Randomized Trial

In a cluster randomized trial, clusters rather than subjects are randomly assigned to a treatment group. Considering centers as clusters, for each center we then have *m*_1*j *_= 0 or *m*_2*j *_= 0. Such a design is also encountered in individually randomized trials in which clustering is imposed by the intervention design and is nested within groups, such as when subjects are assigned to two treatment arms for which the intervention is delivered by several physicians, each participating in only one arm of the study [8,9]. In this case, equation (8) reduces to:(13)

where . With roughly equal cluster sizes and assuming the same number of subjects in each arm (), the *Deff *can be approximated as follows:(14)

where  is the mean cluster size. This value is the inflation factor [4], used for sample size calculation in cluster randomized trials.

##### Multicenter Observational Study

In a multicenter observational study, group sizes are likely to differ, at the level of the center (i.e., *m*_1*j *_≠ *m*_2*j*_) or globally (i.e., *n*_1 _≠ *n*_2_). Nevertheless, with identical group distributions among centers (i.e., the proportion of subjects in group 1 is *p *∈ ]0;1[, whatever the center is), the design effect reduces to:(15)

Thus, in an observational study, with all centers having identical group distributions – even if the global group sizes are not equal (i.e., even if *n*_1 _≠ *n*_2_) – taking into account the center effect leads to increased power, as with stratified individually randomized trials.

*No design effect: Deff = 1*.

From formula (8), *Deff *= 1 leads to:(16)

Rewriting *S *as , we obtain a statistic that estimates, for group 1, the difference between the observed group size (i.e., *m*_1*j*_) and its expected value under the assumption of centers having identical group proportions (i.e., ). Therefore, when this statistic – providing a measure of heterogeneity of the group distributions among centers (thus the level of association between the group and the center) – is below 1, the *Deff *is also below 1 and using a statistical model that takes into account the center effect leads to increased power. On the contrary, when the group distributions differ strongly among centers, the *S *statistic, and then the *Deff*, is greater than 1, thus leading to a loss of power. At the extreme case where centers are totally nested within groups, the loss of power can be very important and it has been shown that omitting the center effect in analyzes leads to type I error [4]. The link between the power of multicenter studies and the design effect can be established as follows. Be *n*_*i *_the size of group *i*, *ES *the expected effect size and *z*_*γ *_the quantile of the standard normal distribution such that P(*Z *≤ *z*_*γ*_) = *γ *(*Z *being *N*(0,1)). The sample size calculation formula allowing testing the group effect on a continuous outcome and corrected for the design effect is [7,10]:(17)

Then, the power of any multicenter study depends on the design effect according to the following relation:(18)

where Φ(·) is defined as the cumulative density function of *N*(0,1). As the design effect increases and exceeds 1, the power decreases and sample size has to be inflated to reach the nominal power. On the contrary, when the design effect value is below 1, the power is larger than the nominal one, allowing reducing the required sample size.

### Example

Table 3 presents data for hypothetical studies of 10 centers of unequal sizes. In each case, the proportion of subjects in group 1 equals 25% but this proportion varies more or less among centers according to the design of the study. The center sizes imbalance is voluntary less important than in the simulation study and represents a more likely study design. This example shows clearly that, when the proportion of subjects in group 1 varies slightly around the global proportion (the "quite homogeneous" column) the design effect is below 1 then indicating a gain in power. On the contrary, when this proportion varies strongly (the "heterogeneous" column), the design effect exceeds 1, involving a loss of power. In the last column, we present the extreme case where centers are nested within the groups. This situation, which can be identified with that of a cluster randomized trial, leads to an important loss in power as shown by the very large design effect.

**Table 3:** Design effects calculations for three different group distributions among centers.

Group distribution among centers	Quite homogeneous			Heterogeneous			Cluster design		
**Group size per center**	*m*_1*j*_	*m*_2*j*_	%*	*m*_1*j*_	*m*_2*j*_	%*	*m*_1*j*_	*m*_2*j*_	%*

Center 1 (n = 57)	16	41	**28**	11	46	**19**	0	57	**0**
Center 2 (n = 38)	10	28	**26**	24	14	**63**	38	0	**100**
Center 3 (n = 44)	11	33	**25**	7	37	**16**	0	44	**0**
Center 4 (n = 15)	3	12	**20**	1	14	**7**	0	15	**0**
Center 5 (n = 41)	9	32	**22**	8	33	**20**	0	41	**0**
Center 6 (n = 19)	5	14	**26**	10	9	**53**	19	0	**100**
Center 7 (n = 37)	8	29	**22**	9	28	**24**	0	37	**0**
Center 8 (n = 52)	12	40	**23**	4	48	**8**	0	52	**0**
Center 9 (n = 12)	3	9	**25**	1	11	**8**	0	12	**0**
Center 10 (n = 28)	8	20	**29**	10	18	**36**	28	0	**100**

*S*	0.14			5.79			33.77		
***Deff *(*ρ *= 0.10)**	**0.91**			**1.48**			**4.28**		

*group 1 proportion in each center

The global proportion of subjects in group 1 is 25%, for each group distribution, and the Intraclass Correlation Coefficient is equal to 0.10.

To illustrate the impact of heterogeneity between the global group sizes on the design effect, we considered hypothetical situations, less likely to occur, where 10 centers recruit 20 subjects each, for balanced designs (i.e., *n*_1 _= *n*_2_, Table S4a in Additional file 1) and imbalanced designs (i.e., *n*_1 _≠ n_2_, Table S4b in Additional file 1), and for different levels of heterogeneity of group distributions among centers and two ICC values. As expected, the *Deff *increases with *S *and increases with the ICC. Moreover, if we focus on the "strongly heterogeneous" column, we observe a higher *Deff *with imbalance between the two groups (Table S4b in Additional file 1, *Deff *= 1.757 for *ρ *= 0.1) than with balance between the groups (Table S4a in Additional file 1, *Deff *= 1.620 for *ρ *= 0.1), which can be analytically explained (Appendix 2). Thus, the impact of heterogeneity of the group distributions among centers is greater with increased imbalance between the two group sizes. See additional file 1 for results from this example.

## Discussion and conclusion

In a multicenter study, the design effect measures the effect of clustering due to multisite recruitment of subjects. As shown in formula (18), the power of such a study is directly affected by the design effect value. Our work aimed explaining why some situations of multicenter studies, such as individually randomized trials, lead to a gain in power whereas others, such as cluster randomized trials lead to a loss of power.

We derived a simple formula assessing the clustering effect in a multicenter study aiming to estimate the effect of a binary factor on a continuous outcome, through an individual level analysis with a mixed effect model: *Deff *= 1+(*S*-1)*ρ*. The design effect depends on *ρ*, the correlation between observations from the same center. It also depends on *S*, a statistic that quantifies the degree of heterogeneity of group distributions among centers, and in other words, the level of association between the binary factor and the center. *S *increases with the heterogeneity of the group distributions among centers, which leads to an increased *Deff *and a loss of power, and falls below 1 when the group distributions are identical between centers, thus leading to a *Deff *below 1 and a gain in power. It is now known that balanced designs such as individually randomized trials increase their power when including the center effect in analyses [5], and that cluster randomized trials should increase their sample size to reach the nominal power and account for the center effect in the analyses to protect against type I error inflation [4]. Our simple formula throws light on the relation between these two situations and allows calculating the design effect for any multicenter design.

We used in our developments a weighted method to assess the group effect: this method gives equal weight to each subject, whatever the size of his/her center is. Different methods of analysis could be used. In the frame of multicenter randomized trials, Lin *et al*. and Senn *et al*. discuss this point and show that a weighted analysis is more powerful than an unweighted one, particularly when there is unbalance in sample sizes between centers [11,12]. The weighted method is then often recommended for analyses of data from multicenter randomized trials, what justifies our choices for model (1) [13]. However, in clusters randomized trials, Kerry *et al*. show that the minimum variance weights are the most efficient weights in the estimation of the design effect in the presence of important imbalance between the clusters sizes, but that weighting the clusters by their sizes give similar – though over estimated – results, except when clusters are large [14]. Our formula aims to apply to any multicenter study, whatever its design is, from individually to cluster randomized trials. Then, it may not use the most powerful method of calculation for some particular multicenter designs but has the great advantage to be simple and general.

Apart from the mixed effect model (1) we described, we did not develop the practical aspect of the analysis stage of a multicenter study. Several statistical software packages are available to perform analyses of correlated data, such as data from multicenter designs. Zhou *et al*. and Murray *et al*. review many of these programs and detail, among others, appropriate procedures and available options allowing specifying data modeling [15,16]. Moreover, some tutorials present step-by-step illustrations of the use of SAS and SPSS mixed model procedures [17,18]. Lastly, Pinheiro and Bates provide an overview of the application of mixed-effects models in S and S-PLUS which are easily transposable to the R software [19].

In the field of cluster randomized trials, several authors worked on the planning of studies through the design effect and sample size calculations and proposed extensions of classical formula, for example to account for imbalance in cluster sizes [20,21]. Our formula does not aim to substitute for these more specific and precise formula but to connect several multicenter designs through a design effect formula. This result helps in understanding the impact of the correlation on power of multicenter studies, whatever their designs are, and is particularly useful for observational studies where the center effect question is not often taken into account at the planning and/or at the analysis stages [22,23]. However, when extended design effects formulas exist, dealing with a particular problem such as that of imbalance cluster sizes in cluster randomized trials, we recommend using them.

This simple result could now be extended to designs including, for example, several nested or crossed levels of correlation. One can then consider cluster-cluster randomization, or cluster then individual randomization and all observational designs including multiple levels of correlation between outcomes. Such designs could bring mixture of gain and loss of power, according to the multiple correlation levels considered. For example, Diehr *et al*. studied the case of matched-pair cluster designs and Giraudeau *et al*. the case of cluster randomized cross-over designs [24,25]. A lot of situations like these ones could be explored to extend our result to more complex designs.

To conclude, clustering of data is a logical consequence of multicenter designs [26,27]. Some designs allow for controlling some factors (e.g., balancing and homogenizing the treatment distribution in individually randomized trials), whereas others exclude such possibility. This latter situation occurs mainly in observational studies, for which there is no way to control the prevalence or distribution of any factor. Since multicenter studies range in design, from homogeneous and balanced designs to "cluster" distribution designs, the design effect can induce a gain or a loss of power as we described. The main advantage of the design effect formula we proposed is its simplicity and its ability to apply to any multicenter study. Its potential weakness would be the difficulty, for an investigator who plans a multicenter study, to obtain an accurate estimate of *S*, the degree of heterogeneity of the group distributions between centers, and of the ICC. In the field of cluster randomized trials, important efforts have been done to improve ICC estimates reporting, which should now be followed for any multicenter study [28,29]. In the same way, recommendations should be made for encouraging the reporting of *Deff *calculation, or of the *S *statistic, from any multicenter study publication. Associated with an ICC estimate, this information could help researchers in planning new multicenter – particularly observational – studies.

## Competing interests

The authors declare that they have no competing interests.

## Authors' contributions

The study was designed by EV and BG. EV performed the statistical analysis and drafted the article, which was then revised by BG. All authors approved the final manuscript.

## Appendix 1

### Calculation of the group effect variance with a two-way ANOVA

In the mixed-effects model (1), the variance of the mean response in group *i *is as follows:

The group effect variance is defined as follows:

Since the centers are independent, we have:

*corr*(*Y*_*ijk*_; *Y*_*i*'*j*'*k*'_) = 0 for *j *≠ *j*' and

*corr*(*Y*_*ijk*_; *Y*_*i*' *jk*'_) = *ρ *for responses from the same center. Then:

which leads to:

## Appendix 2

### Rewriting the S statistic with the between-center group size variances

Assuming centers are of equal sizes, ∀ *j *= 1,..., *Q*,  and we have:

where *V*_1 _is the between-center variance for sizes of group 1. Let  be the mean size for group *i*, then *V*_1 _can be rewritten as follows:

where  is the center size variance and  is the between-center variance for sizes of group 2. Assuming centers are of equal sizes, we have ∀ *j *= 1,..., *Q*, ; thus *V*_*m *_= 0 and *V*_1 _= *V*_2_. The statistic is then:

Hence, assuming centers are of equal sizes, for a given total sample size *N*, number of centers *Q*, and between-center group size variance *V*_*i*_, the higher the difference between  and 1 the higher the statistic *S*. Then, the *Deff *increases with the degree of imbalance between the two group sizes. This result generalizes to designs with unequal center sizes, because the *S *statistic always depends on . However, quantitative prediction of the impact of the  ratio on the *Deff *is not straightforward because the center size variance, *V*_*m*_, and the covariance term between *V*_*m *_and *V*_2 _are, in this case, not null.

## Pre-publication history

The pre-publication history for this paper can be accessed here:

http://www.biomedcentral.com/1471-2288/9/39/prepub

## Supplementary Material

Additional file 1 **Table S4 – Design effects calculations for hypothetical studies with different group distributions among centers**. These are tables S4a and S4b, illustrating the last example presented in the manuscript. Tables present design effects calculations for different study designs, different group distributions among centers, two Intraclass Correlation Coefficient values and for balanced and imbalanced designs. Click here for file
