# Supplementary material for: Design effect in multicenter studies: gain or loss of power?
Source: BMC Med Res Methodol. 2009 Jun 18;9:39. doi: 10.1186/1471-2288-9-39 (PMC2715424; doi:10.1186/1471-2288-9-39)
Supplement: Additional file 1 — Table S4 – Design effects calculations for hypothetical studies with different group distributions among centers. These are tables S4a and S4b, illustrating the last example presented in the manuscript. Tables present design effects calculations for different study designs, different group distributions among centers, two Intraclass Correlation Coefficient values and for balanced and imbalanced designs. [file 1471-2288-9-39-S1.pdf]

Table 4: Design effects calculations for hypothetical studies with different group distributions among centers.

4.a. Balanced designs ($n_1 = n_2$) with $N = 200$ subjects and $Q = 10$ centers of equal size (case of randomized trials with two treatment arms).

**Group distribution**	**Homogeneous**		**Slightly heterogeneous**		**$S^* = 1$**		**Heterogeneous**		**Strongly heterogeneous**		**Cluster design**	
Group sizes per center	m_{1j}	m_{2j}	m_{1j}	m_{2j}	m_{1j}	m_{2j}	m_{1j}	m_{2j}	m_{1j}	m_{2j}	m_{1j}	m_{2j}
Center 1	10	10	8	12	6	14	14	6	17	3	20	0
Center 2	10	10	11	9	12	8	8	12	6	14	0	20
Center 3	10	10	10	10	10	10	11	9	12	8	20	0
Center 4	10	10	11	9	11	9	6	14	4	16	0	20
Center 5	10	10	9	11	9	11	12	8	16	4	20	0
Center 6	10	10	11	9	13	7	5	15	3	17	0	20
Center 7	10	10	10	10	9	11	15	5	14	6	20	0
Center 8	10	10	10	10	10	10	7	13	2	18	0	20
Center 9	10	10	8	12	7	13	12	8	19	1	20	0
Center 10	10	10	12	8	13	7	10	10	7	13	0	20
S^*	0		0.32		1		2.08		7.20		20	
$\frac{n_1 n_2}{NQ} S^*$	0		1.6		5		10.4		36		100	
$Deff^{**}$ ($\rho = 0.01$)	0.990		0.993		1.000		1.011		1.062		1.190	
$Deff^{**}$ ($\rho = 0.1$)	0.900		0.932		1.000		1.108		1.620		2.900	

m_{ij} : size of group i in center j. * $S = \frac{N}{n_1 n_2} \sum_{j=1}^{Q} \left(m_{1j} - \frac{m_j n_1}{N} \right)^2$. ** Design effect.

4.b. Imbalanced designs ($n_1 = 140$, $n_2 = 60$) with $N = 200$ subjects and $Q = 10$ centers of equal size (case of observational studies with a binary risk factor).

**Group distribution**	**Homogeneous**		**Slightly heterogeneous**		**$S^* = 1$**		**Heterogeneous**		**Strongly heterogeneous**	
Group sizes per center	m_{1j}	m_{2j}	m_{1j}	m_{2j}	m_{1j}	m_{2j}	m_{1j}	m_{2j}	m_{1j}	m_{2j}
Center 1	14	6	15	5	14	6	18	2	20	0
Center 2	14	6	13	7	12	8	9	11	20	0
Center 3	14	6	14	6	16	4	13	7	8	12
Center 4	14	6	15	5	12	8	16	4	8	12
Center 5	14	6	16	4	17	3	12	8	20	0
Center 6	14	6	13	7	11	9	11	9	20	0
Center 7	14	6	12	8	16	4	16	4	20	0
Center 8	14	6	14	6	12	8	10	10	8	12
Center 9	14	6	15	5	14	6	17	3	8	12
Center 10	14	6	13	7	16	4	18	2	8	12
S^*	0		0.33		1		2.48		8.57	
$\frac{n_1 n_2}{NQ} S^*$	0		1.4		4.2		10.4		36	
$Deff^{**}$ ($\rho = 0.01$)	0.990		0.993		1.000		1.015		1.076	
$Deff^{**}$ ($\rho = 0.1$)	0.900		0.933		1.000		1.148		1.757	

m_{ij} : size of group i in center j. * $S = \frac{N}{n_1 n_2} \sum_{j=1}^{Q} \left(m_{1j} - \frac{m_j n_1}{N} \right)^2$. ** Design effect.
